# Is compromised intestinal barrier integrity responsible for the poor prognosis in critically ill patients with pre-existing hyperglycemia?

**DOI:** 10.1186/s13098-022-00943-5

**Published:** 2022-11-17

**Authors:** Yi-Feng Wang, Feng-Ming Liang, Min Liu, Li-Cheng Ding, Jiao-Jie Hui, Hong-Yang Xu, Li-Jun Liu

**Affiliations:** 1 Department of Critical Care Medicine, The Affiliated Wuxi People’s Hospital of Nanjing Medical University, Wuxi, China; 2 Department of Emergency Medicine, Affiliated Hospital of Jiangnan University, Wuxi, China; 3 Department of Emergency and Critical Care Medicine, Second Affiliated Hospital of Soochow University, Suzhou, China

**Keywords:** Intestinal barrier, Hyperglycemia, Critical illness, Prognosis

## Abstract

**Background:**

Compromised intestinal barrier integrity can be independently driven by hyperglycemia, and both hyperglycemia and intestinal barrier injury are associated with poor prognosis in critical illness. This study investigated the intestinal barrier biomarkers in critically ill patients, to explore the role of compromised intestinal barrier integrity on the prognosis of critically ill patients with pre-existing hyperglycemia.

**Methods:**

This was a retrospective observational study. The relationships between intestinal barrier biomarkers and glycated hemoglobin A1c (HbA1c), fasting blood glucose (FBG), indicators of clinical characteristics, disease severity, and prognosis in critically ill patients were investigated. Then the metrics mentioned above were compared between survivors and non-survivors, the risk factors of 90-day mortality were investigated by logistic regression analysis. Further, patients were divided into HbA1c < 6.5% Group and HbA1c ≥ 6.5% Group, metrics mentioned above were compared between these two groups.

**Results:**

A total of 109 patients with critical illness were included in the study. D-lactate and lipopolysaccharide (LPS) were associated with sequential organ failure assessment (SOFA) score and 90-day mortality. LPS was an independent risk factor of 90-day mortality. DAO, NEU (neutrophil) proportion, temperature, lactate were lower in HbA1c ≥ 6.5% Group while D-lactate, LPS, indicators of disease severity and prognosis showed no statistical difference between HbA1c < 6.5% Group and HbA1c ≥ 6.5% Group.

**Conclusions:**

Intestinal barrier integrity is associated with the disease severity and prognosis in critical illness. Compromised intestinal barrier integrity might be responsible for the poor prognosis in critically ill patients with pre-existing hyperglycemia.

## Background

Diabetes has become a huge burden on global health, and the worldwide prevalence of diabetes in adults in 2021 was estimated to be 10.5% [1]. As one of the most common comorbidities, diabetes is strongly associated with poor prognosis in critical illness [2, 3]. As is known to all, hyperglycemia is the most specific symptom of diabetes, and recent studies have updated our knowledge that hyperglycemia, irrespective of the diabetes status, is associated with increased risk of in-hospital mortality in critically ill patients [4, 5]. However, the underlying mechanism by which hyperglycemia results in poor prognosis remains debated.

In addition to hyperglycemia, another condition frequently happened in critical illness is intestinal barrier injury. Intestinal barrier consists of mucosal barrier, biological barrier, and immune barrier, preventing bacteria and toxins crossing the barrier from the intestinal tract to the circulation during nutrition absorption, and the impairment of intestinal barrier facilitating the translocation of intestinal bacteria and their metabolites [6]. Nowadays, the compromised intestinal barrier integrity and associated bacterial translocation have been recognized as causative factors of poor prognosis in critical illness [7, 8].

Interestingly, the compromised intestinal barrier integrity and bacterial translocation are closely related to hyperglycemia [9–11]. Preclinical study has confirmed that hyperglycemia can independently drive the impairment of intestinal barrier, facilitate intestinal bacteria translocation and increase the risk of enteric infection [12]. Our clinical study has found that the impairment of intestinal barrier got worse with the prolongation of hyperglycemia [13]. Recently, the compromised intestinal barrier integrity in COVID-19 has been well reviewed that predisposes patients with pre-existing hyperglycemia to aggravated endotoxemia and finally worse outcome [14]. Considering the co-result of critical illness and hyperglycemia, we assumed that the compromised intestinal barrier integrity might be responsible for the poor prognosis in critically ill patients with pre-existing hyperglycemia.

In the present study, we conducted a retrospective observational study, investigated the intestinal barrier biomarkers in critically ill patients, to explore the roles of compromised intestinal barrier integrity on the prognosis of critically ill patients with pre-existing hyperglycemia.

## Methods

### Study population

In the present study, we reviewed the medical data of all patients admitted to the Department of Critical Care Medicine of the Affiliated Wuxi People's Hospital of Nanjing Medical University from June 2017 to December 2018. The inclusion criteria were patients who were older than 18 years of age and the exclusion criteria were patients who were unable to get complete medical records, receiving immunosuppressive therapy (eg, immunosuppressants, high-dose glucocorticoids, chemotherapy, and radiotherapy), had diseases with impaired immunity (eg, carcinoma, leukemia, lymphoma, and AIDS), had intestinal diseases (eg, inflammatory bowel disease, intestinal obstruction, and mesenteric vascular obstruction), had gastrointestinal surgery history within a year, had an altered relationship between glycated hemoglobin A1c (HbA1c) and glycemia (eg, massive blood transfusion after life-threatening hemorrhage, hemodialysis, and erythropoietin therapy) [15].

### Data collection

The clinical data were collected from the hospital information system including (i) medical history and demographics (age and sex); (ii) HbA1c, fasting blood glucose (FBG), intestinal barrier biomarkers [diamine oxidase (DAO), D-lactate, and lipopolysaccharide (LPS)], metrics of routine biochemical test, dosage of norepinephrine (NE), acute physiology and chronic health evaluation II (APACHE II) score, and sequential organ failure assessment (SOFA) score within first 24 h. For those with more than one result, the worst ones were recorded; (iii) length of stay (LOS) in hospital, LOS in ICU, and 90-day mortality.

### Intestinal barrier biomarkers

The concentrations of DAO, D-lactate, and LPS in plasma were detected using the Diamine Oxidase, Lactic Acid, Bacterial Endotoxin Assay Kit (Enzyme, Zhong Sheng Jinyu Diagnostic Technology Co., Ltd. Beijing, China) in JY-Po-Color DLT Set.

Although all the three biomarkers can indicate the impairment of intestinal barrier, they represents different part of intestinal barrier injury. DAO is abundantly expressed in IECs and the elevated DAO in circulation reflects the damage of IECs [16]. And as the metabolites of bacteria, elevated D-lactate and LPS in circulation imply the compromised intestinal barrier integrity as well as increased bacterial translocation [17].

### HbA1c

Blood HbA1c levels were measured upon ICU admission using Variant II analyzer (Bio Rad, Hercules, CA). HbA1c reflects the average blood glucose level in the past 2 to 3 months, and HbA1c ≥ 6.5 is one of the criteria for the diagnosis of diabetes [15]. Although stress-induced hyperglycemia is common in critically ill patients, Luethi et al. have found that HbA1c quantified at ICU admission has not been altered by the onset of critical illness, making HbA1c a reliable indicator for the chronic glycemic control [18].

### Statistical method

Sample size was not determined by statistical calculation, but equal to the number of patients with available records of intestinal barrier biomarkers and HbA1c during the study period. SPSS 25 (IBM Corporation, Chicago, IL, USA) was used for statistical analysis. Kolmogorov–Smirnov tests were performed to determine the normality of continuous variables. Normal distribution variables were expressed as mean ± standard deviation while non-normal distribution variables were expressed as median (interquartile range). Relationships between two continuous variables were assessed by Spearman correlation coefficient. Differences of normal distribution variables between two groups were assessed by Student’s t test while differences of non-normal distribution variables between two groups were assessed by Mann–Whitney test. Categorical data were assessed by Chi-square or Fisher’s exact test. Risk factors for 90-day mortality were investigated by logistic regression, variables with a P value < 0.05 in the univariate logistic analysis were included in the followed multivariate logistic analysis. All statistical tests were 2-tailed, and statistical significance was set at a 5% level.

## Results

### Intestinal barrier biomarkers, HbA1c, FBG, indicators of clinical characteristics, disease severity, and prognosis in all patients

A total of 109 patients with critical illness were included in the study. The main reasons for ICU admission were trauma (n = 24), sepsis (n = 23), respiratory failure (n = 19), cerebrovascular accident (n = 14), MODS (n = 12), cardiac arrest (n = 7), heatstroke (n = 6), heart failure (n = 4). The intestinal barrier biomarkers, HbA1c, FBG, indicators of clinical characteristics, disease severity, and prognosis in all patients were shown in Table 1.

**Table 1:** The basic information

Demographics	
Sex (male/female)	66/43
Age, y	65 (45, 76.5)
HbA1c, %	5.9 (5.2, 6.85)
FBG, mmol/L	7.67 (6.11, 10.24)
Intestinal barrier biomarkers	
DAO, U/L	24.1 (10.11, 37.1)
D-lactate, mg/L	35.83 (19.25, 52.72)
LPS, U/L	10.31 (6.28, 18.85)
Infection and immunity	
WBC, 10^9/L	15.14 ± 7.72
NEU proportion, %	91.1 (88.0, 93.9)
CRP, mg/L	70.1 (16.75, 172.8)
Procalcitonin, µg/L	2.05 (0.66, 9.32)
HLA-DR, %	45.45 (30.43, 63.65)
Temperature, ℃	38.2 (37.45, 38.75)
Respiratory and circulatory system	
Oxygenation index	260 (197, 340)
Respiratory rate, RPM	23 (19.5, 25)
Heart rate, RPM	108 (88, 122)
MAP, mmHg	66.67 (60.67, 81.33)
Artery blood pH	7.36 (7.27, 7.41)
Lactate, mmol/L	3.2 (1.8, 5.1)
Dosage of NE, ug/kg/min	0.13 (0, 0.43)
Renal function	
Urea nitrogen, mmol/L	8.2 (5.35, 11.5)
Creatinine, µmol/L	105.8 (72.25, 166.9)
AKI morbidity (N)	35.8% (39)
Liver function	
ALT, U/L	38 (18.5, 87)
AST, U/L	45 (27.5, 92)
Total bilirubin, µmol/L	20.4 (12.95, 32.05)
Direct bilirubin, µmol/L	10.2 (6.3, 17.45)
Total protein, g/L	51.42 ± 12.96
Albumin, g/L	28.01 ± 7.91
Prealbumin, mg/L	148.76 ± 82.48
Disease severity and prognosis	
APACHE II	16 (11, 21)
SOFA	7 (6, 9.5)
LOS in hospital, day	22 (12, 40.5)
LOS in ICU, day	6 (3, 14)
90-day mortality (N)	17.6% (19)

*HbA1c* hemoglobin A1c, *FBG* fasting blood glucose, *DAO* diamine oxidase, *LPS* lipopolysaccharide, *WBC* white blood cell, *NEU* neutrophil, *CRP* C-reactive protein, *RPM* rate per minute, *MAP* mean arterial pressure, *NE* norepinephrine, *AKI* acute kidney injury, *ALT* alanine transaminase, *AST* aspartate transaminase, *APACHE II* acute physiology and chronic health evaluation II, *SOFA* sequential organ failure assessment, LOS length of stay

**Fig. 1:**
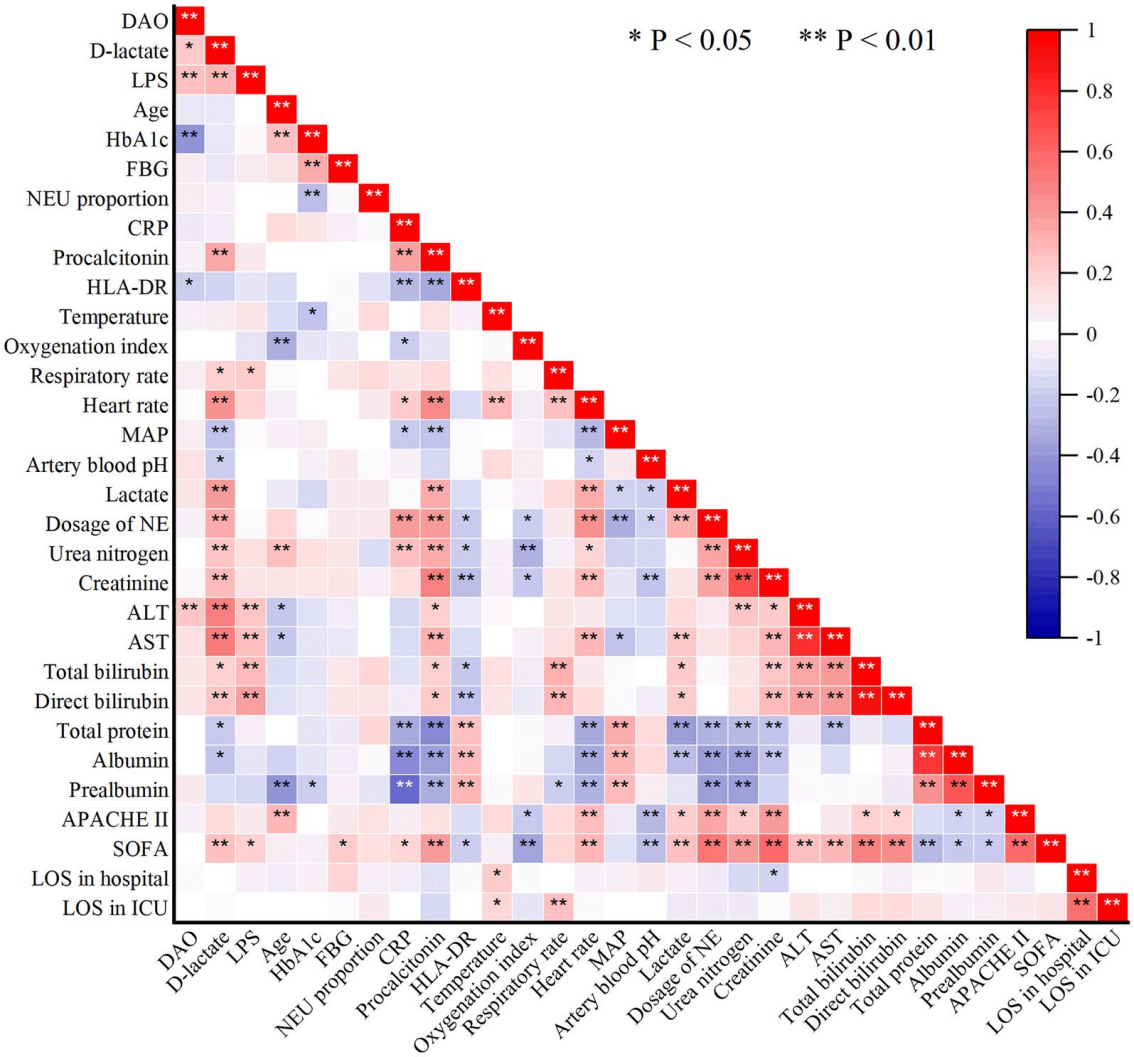
Correlation analyses between intestinal barrier biomarkers and other clinical indicators

The correlation between these metrics mentioned above were shown in Table 2 and Fig. 1. While DAO, D-lactate and LPS were positively correlated with each other, their correlations with other metrics were not exactly the same. DAO was negatively correlated with HbA1c and HLA-DR, and positively correlated with alanine transaminase (ALT). D-lactate was positively correlated with procalcitonin, respiratory rate, heart rate, lactate, dosage of NE, urea nitrogen, creatinine, ALT, aspartate transaminase (AST), total bilirubin and direct bilirubin, while negatively correlated with MAP, artery blood pH, total protein and albumin. LPS was positively correlated with respiratory rate and majority indicators of liver function. When it came to the indicators of disease severity and prognosis, both D-lactate and LPS were associated with SOFA score and 90-day mortality.

**Table 2:** Correlation analyses between intestinal barrier biomarkers, HbA1c, FGB, indicators of clinical characteristics, disease severity, and prognosis

	DAO		D-lactate		LPS	
	r value	P value	r value	P value	r value	P value
Intestinal barrier biomarkers						
DAO	–	–	0.223	0.020*	0.279	0.003**
D-lactate	0.223	0.020*	–	–	0.305	0.001**
LPS	0.279	0.003**	0.305	0.001**	–	–
Demographics						
Sex	–	0.484	–	0.938	–	0.209
Age	− 0.083	0.394	− 0.087	0.366	0.032	0.740
HbA1c	− 0.436	< 0.001**	− 0.087	0.370	− 0.038	0.695
FBG	0.092	0.341	− 0.086	0.376	0.099	0.307
Infection and immunity						
WBC	− 0.045	0.640	0.036	0.707	− 0.075	0.440
NEU proportion	0.096	0.325	− 0.046	0.637	0.005	0.962
CRP	− 0.090	0.353	− 0.076	0.434	0.031	0.750
Procalcitonin	0.064	0.511	0.366	< 0.001**	0.106	0.271
HLA-DR	− 0.219	0.027*	− 0.182	0.067	− 0.109	0.274
Temperature	0.062	0.522	0.084	0.385	0.133	0.169
Respiratory and circulatory system						
Oxygenation index	0.013	0.890	0.002	0.983	− 0.108	0.264
Respiratory rate	0.092	0.343	0.216	0.024*	0.221	0.021*
Heart rate	0.043	0.660	0.451	< 0.001**	0.181	0.060
MAP	0.086	0.376	− 0.248	0.009**	0.044	0.646
Artery blood pH	0.150	0.120	− 0.202	0.035*	0.030	0.759
Lactate	0.139	0.152	0.418	< 0.001**	0.019	0.844
Dosage of NE	0.061	0.529	0.356	< 0.001**	0.058	0.548
Renal function						
Urea nitrogen	− 0.004	0.965	0.247	0.010**	0.143	0.137
Creatinine	0.040	0.679	0.287	0.003**	0.121	0.211
AKI morbidity	–	0.226	–	0.016*	–	0.069
Liver function						
ALT	0.257	0.007**	0.502	< 0.001**	0.257	0.007**
AST	0.157	0.103	0.531	< 0.001**	0.266	0.005**
Total bilirubin	0.125	0.195	0.204	0.034*	0.293	0.002**
Direct bilirubin	0.135	0.161	0.259	0.007**	0.398	< 0.001**
Total protein	0.013	0.893	− 0.237	0.013*	− 0.047	0.626
Albumin	− 0.008	0.936	− 0.242	0.011*	− 0.109	0.260
Prealbumin	0.117	0.225	− 0.144	0.134	− 0.173	0.072
Disease severity and prognosis						
APACHE II	0.060	0.533	0.168	0.081	0.109	0.261
SOFA	0.016	0.866	0.277	0.004**	0.205	0.033*
LOS in hospital	0.055	0.567	− 0.006	0.953	− 0.047	0.626
LOS in ICU	− 0.019	0.842	0.041	0.670	− 0.012	0.898
90-day mortality	–	0.905	–	0.038*	–	0.027*

*HbA1c* hemoglobin A1c, *FBG* fasting blood glucose, *DAO* diamine oxidase, *LPS* lipopolysaccharide, *WBC* white blood cell, *NEU* neutrophil, *CRP* C-reactive protein, *RPM* rate per minute, *MAP* mean arterial pressure, *NE* norepinephrine, *AKI* acute kidney injury, *ALT* alanine transaminase, *AST* aspartate transaminase, *APACHE II* acute physiology and chronic health evaluation II, *SOFA* sequential organ failure assessment, LOS length of stay

^*^P < 0.05, **P < 0.01

### Relationships between intestinal barrier biomarkers, disease severity, and prognosis in all patients

Then we compared the intestinal barrier biomarkers and other clinical indicators between survivors and non-survivors (Table 3). The results showed that D-lactate, LPS, procalcitonin, lactate, dosage of NE, urea nitrogen, creatinine, acute kidney injury (AKI) morbidity, APACHE II score, and SOFA score were higher, while prealbumin, LOS in hospital, and LOS in ICU were lower in non-survivors.

**Table 3:** The comparisons of intestinal barrier biomarkers, HbA1c, FBG, indicators of clinical characteristics, disease severity, and prognosis between survivors and non-survivors

	Survivors	Non-survivors		P value
Demographics				
Sex (male/female)	51/39	15/4		0.119
Age, y	63.5 (44.75, 76.25)	66 (55, 79)		0.531
HbA1c, %	5.75 (5.2, 6.83)	6.1 (5.4, 7)		0.522
FBG, mmol/L	7.88 (6.11, 10.40)	7.35 (5.95, 8.55)		0.554
Intestinal barrier biomarkers				
DAO, U/L	24.1 (10.50, 33.8)	28.51 (8.54, 37.34)		0.905
D-lactate, mg/L	33.79 (18.48, 49.95)	47.54 (27.52, 60.58)		0.038*
LPS, U/L	9.36 (6.19, 15.27)	17.73 (7.96, 26.49)		0.027*
Infection and immunity				
WBC, 10^9/L	14.51 ± 7.03	18.12 ± 10.10		0.178
NEU proportion, %	91 (88.2, 94)	92.2 (83.6, 93.7)		0.806
CRP, mg/L	65.95 (17.38, 166.8)	96.9 (10.3, 197.1)		0.669
Procalcitonin, µg/L	1.76 (0.47, 8.37)	4.16 (1.87, 24.17)		0.045*
HLA-DR, %	46.1 (34.25, 66.68)	30.65 (26.25, 57.83)		0.076
Temperature, ℃	38.2 (37.5, 38.8)	38.0 (37.0, 38.7)		0.316
Respiratory and circulatory system				
Oxygenation index	258 (197, 340)	268 (194, 343)		0.618
Respiratory rate, RPM	23 (20, 25)	23 (16, 25)		0.418
Heart rate, RPM	107.5 (86.25, 122)	108 (92, 130)		0.352
MAP, mmHg	67 (60.9, 79)	65 (59.3, 101.3)		0.917
Artery blood pH	7.37 (7.27, 7.41)	7.33 (7.23, 7.42)		0.417
Lactate, mmol/L	3.0 (1.6, 4.9)	4.1 (2.9, 9.2)		0.019*
Dosage of NE, ug/kg/min	0.10 (0, 0.30)	0.60 (0.10, 1.27)		0.004**
Renal function				
Urea nitrogen, mmol/L	7.85 (4.98, 10.63)	10.4 (6.6, 18.8)		0.017*
Creatinine, µmol/L	102.2 (70.78, 151.6)	150.9 (89.7, 271.8)		0.039*
AKI morbidity (N)	31.1% (28)	57.9% (11)		0.027*
Liver function				
ALT, U/L	39 (19, 72)	32 (17, 328)		0.528
AST, U/L	43.5 (27, 83)	59 (30, 220)		0.096
Total bilirubin, µmol/L	18.3 (12.63, 29.85)	24.7 (14.6, 39.3)		0.128
Direct bilirubin, µmol/L	9.75 (6.05, 16.85)	13.3 (7.0, 25.3)		0.174
Total protein, g/L	52.42 ± 12.69	46.64 ± 13.54		0.084
Albumin, g/L	28.61 ± 7.68	25.13 ± 8.52		0.090
Prealbumin, mg/L	156.97 ± 81.69	109.86 ± 76.73		0.017*
Disease severity and prognosis				
APACHE II	15 (10, 19.25)	20 (17, 24)		< 0.001**
SOFA	7 (5, 9)	9 (9, 12)		0.001**
LOS in hospital, day	25.5 (13, 41.5)	13 (3, 26)		0.002**
LOS in ICU, day	7 (3, 15.8)	3 (2, 6)		0.015*

*HbA1c* hemoglobin A1c, *FBG* fasting blood glucose, *DAO* diamine oxidase, *LPS* lipopolysaccharide, *WBC* white blood cell, *NEU* neutrophil, *CRP* C-reactive protein, *RPM* rate per minute, *MAP* mean arterial pressure, *NE* norepinephrine, *AKI* acute kidney injury, *ALT* alanine transaminase, *AST* aspartate transaminase, *APACHE II* acute physiology and chronic health evaluation II, *SOFA* sequential organ failure assessment, LOS length of stay

^*^P < 0.05, ** P < 0.01

Further, the risk factors of 90-day mortality were investigated by logistic regression analysis (Table 4). Considering the close relationship with mortality, indicators of disease severity and prognosis were not included in the analysis. The univariate logistic regression analyses showed that D-lactate, LPS, lactate, dosage of NE, urea nitrogen, creatinine, AKI morbidity, ALT, prealbumin, were the risk factors of 90-day mortality (P < 0.05). Then these variables were included in the followed multivariate logistic regression analysis, and the results indicated that LPS was an independent risk factor of 90-day mortality.

**Table 4:** Risk factors associated with 90-day mortality in critically ill patients

Univariate analysis		OR	95% CI	P value
Demographics				
Sex (male/female)		0.349	0.107–1.134	0.080
Age		1.009	0.983–1.036	0.492
HbA1c		1.035	0.760–1.409	0.826
FBG		0.977	0.866–1.102	0.707
Intestinal barrier biomarkers				
DAO		1.001	0.972–1.030	0.968
D-lactate		1.030	1.002–1.060	0.038*
LPS		1.057	1.015–1.101	0.008**
Infection and immunity				
WBC		1.059	0.995–1.127	0.070
NEU proportion		0.978	0.902–1.060	0.587
CRP		1.002	0.996–1.008	0.485
Procalcitonin		1.009	0.996–1.022	0.164
HLA-DR		0.977	0.951–1.004	0.090
Temperature		0.787	0.572–1.084	0.143
Respiratory and circulatory system				
Oxygenation index		1.001	0.996–1.005	0.735
Respiratory rate		0.952	0.875–1.035	0.252
Heart rate		1.010	0.994–1.026	0.224
MAP		1.005	0.983–1.027	0.673
Artery blood pH		0.168	0.010–2.953	0.223
Lactate		1.147	1.026–1.283	0.016*
Dosage of NE		2.396	1.173–4.895	0.017*
Renal function				
Urea nitrogen		1.092	1.019–1.169	0.012*
Creatinine		1.007	1.002–1.012	0.010*
AKI morbidity		3.045	1.104–8.394	0.031*
Liver function				
ALT		1.001	1.000–1.002	0.042*
AST		1.001	1.000–1.002	0.146
Total bilirubin		1.016	0.997–1.034	0.098
Direct bilirubin		1.025	0.998–1.052	0.066
Total protein		0.962	0.922–1.004	0.078
Albumin		0.943	0.882–1.008	0.084
Prealbumin		0.992	0.984–0.999	0.027*

Multivariate analysis	OR	95% CI	P value
Multivariate 1			
LPS	1.056	1.014–1.100	0.009**
Multivariate 2			
LPS	1.057	1.013–1.104	0.011*
Dosage of NE	2.354	1.136–4.875	0.021*

*OR* odds ratio, *CI* confidence interval, *HbA1c* hemoglobin A1c, *FBG* fasting blood glucose, *DAO* diamine oxidase, *LPS* lipopolysaccharide, *WBC* white blood cell, *NEU* neutrophil, *CRP* C-reactive protein, *MAP* mean arterial pressure, *NE* norepinephrine, *AKI* acute kidney injury, *ALT* alanine transaminase, *AST* aspartate transaminase

^*^ P < 0.05, ** P < 0.01

### The intestinal barrier biomarkers and other clinical indicators between patients with and without pre-existing hyperglycemia

According to the results of HbA1c, patients were divided into HbA1c < 6.5% Group and HbA1c ≥ 6.5% Group, the intestinal barrier biomarkers and other clinical indicators were compared between these two groups (Table 5). The results showed that DAO was lower in HbA1c ≥ 6.5% Group but neither D-lactate nor LPS was statistically different between these two groups. Among other clinical indicators, HbA1c ≥ 6.5% Group was higher in the age and urea nitrogen but lower in the neutrophil (NEU) proportion, temperature, lactate, and prealbumin. Besides, the sex composition of two groups was different. As to the indicators of disease severity and prognosis, the differences between two groups was not statistically significant.

**Table 5:** The comparisons of intestinal barrier markers, HbA1c, FBG, indicators of clinical characteristics, disease severity, and prognosis between patients with and without persistent hyperglycemia

	HbA1c < 6.5 Group	HbA1c ≥ 6.5 Group	P value
Demographics			
Sex (male/female)	53/23	13/20	0.003**
Age, y	55.5 (41.25, 74.25)	71 (63, 79)	0.005**
HbA1c, %	5.4 (5.1, 5.9)	7.5 (6.9, 9.35)	< 0.001**
FBG, mmol/L	7.14 (5.61, 8.40)	10.54 (7.75, 14.69)	< 0.001**
Intestinal barrier markers			
DAO, U/L	25.67 (13.17, 40.7)	21.06 (7.98, 29.43)	0.043*
D-lactate, mg/L	36.32 (18.13, 53.23)	34.08 (21.15, 48.75)	0.817
LPS, U/L	9.99 (6.01, 18.95)	10.43 (7.33, 16.99)	0.712
Infection and immunity			
WBC, 10^9/L	15.08 ± 7.47	15.29 ± 8.40	0.896
NEU proportion, %	92.0 (88.4, 94.2)	89.05 (86.55, 91.5)	0.027*
CRP, mg/L	65.35 (14.63, 131.3)	81.9 (18.25, 206.7)	0.222
Procalcitonin, µg/L	2.12 (0.70, 9.56)	2.05 (0.49, 8.87)	0.828
HLA-DR, %	47.1 (30.2, 62.7)	40.2 (30.5, 69.8)	0.749
Temperature, ℃	38.4 (37.5, 39)	37.7 (37.1, 38.6)	0.043*
Respiratory and circulatory system			
Oxygenation index	268 (200, 340)	252 (174, 332)	0.207
Respiratory rate, RPM	23 (20, 25)	22 (16.5, 25)	0.465
Heart rate, RPM	107.5 (87.25, 122)	110 (88, 122)	0.704
MAP, mmHg	66.17 (60, 90.17)	67.67 (63.83, 77)	0.516
Artery blood pH	7.37 (7.28, 7.40)	7.34 (7.24, 7.46)	0.520
Lactate, mmol/L	3.6 (2.0, 5.5)	2.5 (1.5, 4.45)	0.044*
Dosage of NE, ug/kg/min	0.12 (0, 0.50)	0.15 (0, 0.27)	0.992
Renal function			
Urea nitrogen, mmol/L	7.8 (4.58, 10.7)	9.1 (6.85, 13.1)	0.031*
Creatinine, µmol/L	97.45 (69.33, 150.6)	123 (81.35, 196.5)	0.145
AKI morbidity (N)	32.9% (25)	42.4% (14)	0.340
Liver function			
ALT, U/L	39 (19.25, 96)	32 (18, 74)	0.499
AST, U/L	45.5 (30, 106)	37 (24.5, 77)	0.131
Total bilirubin, µmol/L	21.1 (12.93, 33.28)	16.9 (13.05, 31.05)	0.573
Direct bilirubin, µmol/L	10.35 (6.13, 17.38)	8.6 (6.4, 19.55)	0.934
Total protein, g/L	51.87 ± 13.19	50.38 ± 12.55	0.584
Albumin, g/L	28.34 ± 8.10	27.23 ± 7.49	0.501
Prealbumin, mg/L	159.95 ± 87.16	122.99 ± 64.59	0.016*
Disease severity and prognosis			
APACHE II	15 (10, 20.75)	17 (13.5, 21)	0.288
SOFA	7 (5.25, 9)	8 (7, 10)	0.287
LOS in hospital, day	22 (11.25, 39)	23 (14.5, 46)	0.285
LOS in ICU, day	5.5 (3, 14.5)	6 (3, 13)	0.879
90-day mortality (N)	18.4% (14)	15.2% (5)	0.679

*HbA1c* hemoglobin A1c, *FBG* fasting blood glucose, *DAO* diamine oxidase, *LPS* lipopolysaccharide, *WBC* white blood cell, *NEU* neutrophil, *CRP* C-reactive protein, *RPM* rate per minute, *MAP* mean arterial pressure, *NE* norepinephrine, *AKI* acute kidney injury, *ALT* alanine transaminase, *AST* aspartate transaminase, *APACHE II* acute physiology and chronic health evaluation II, *SOFA* sequential organ failure assessment, LOS length of stay

^*^ P < 0.05, ** P < 0.01

## Discussion

In the present study, we investigated the relationships between intestinal barrier biomarkers, HbA1c, FBG, indicators of clinical characteristics, disease severity, and prognosis in critically ill patients. The results showed that D-lactate and LPS were associated with SOFA score and 90-day mortality, LPS was an independent risk factor of 90-day mortality. Additionally, the diversities of intestinal barrier biomarkers and clinical indicators between HbA1c < 6.5% Group and HbA1c ≥ 6.5% Group provided some clues about the roles of compromised intestinal barrier integrity on the prognosis of critically ill patients with pre-existing hyperglycemia.

### The effect of compromised intestinal barrier integrity on disease severity and prognosis in critical illnes

In the present study, three intestinal barrier biomarkers (DAO, D-lactate, and LPS) were used to indicate the different parts of intestinal barrier injury, that DAO reflected the IECs damage [16], while D-lactate and LPS implied the compromised intestinal barrier integrity [17]. Under the severe pathophysiology challenges of critical illness, intestinal barrier injury accompanied with compromised intestinal barrier integrity commonly exists in critically ill patients, causing bacterial translocation, systemic inflammatory response, malabsorption, and consequently the poor prognosis [7, 8, 14]. Accordingly, intestinal barrier biomarkers can be used to predict the prognosis of critical illness [19], and D-lactate has been used in the prognosis of critically ill patients in Qiu’s study [20]. Consisting with previous studies, our study found that D-lactate and LPS were associated with SOFA score and 90-day mortality. As an indicator of disease severity and prognosis, SOFA score employs six metrics reflecting the function of each organ system (respiratory, circulatory, renal, liver, neurological, and haematological) [21]. In our study, the association between SOFA score and the biomarkers of compromised intestinal barrier integrity were also supported by the relationships between the biomarkers and the indicators of clinical characteristics, that D-lactate was correlated with most metrics of respiratory, circulatory, renal and liver function while LPS was correlated with respiratory rate and majority metrics of liver function. Additionally, logistic regression analysis showed that LPS was an independent risk factor of 90-day mortality. All these results suggested that the intestinal barrier integrity was associated with the disease severity and prognosis in critical illness.

Interestingly, the performances of D-lactate and LPS were distinct with DAO in our study. In other words, the compromised intestinal barrier integrity did not accompanied with the damage of IECs. The asynchrony of intestinal barrier biomarkers can be partly explained by the involvement of gut dysbiosis. As the main part of intestinal biological barrier, gut microbiota is significant for the integrity and function of intestinal barrier, and yet the dysbiosis of gut microbiota, also known as gut dysbiosis, will impair the homeostatic balance of intestinal barrier integrity [6]. Clinical study has found that gut microbiota is associated with 28-day mortality among critically ill patients [22]. Given the interactions with various organs, the gut dysbiosis and compromised intestinal barrier integrity deeply participate in the development and exacerbation of critical illness [23–25]. Since the gut dysbiosis has profound effects on the development, maintenance, and outcomes of sepsis [26], the different performances of D-lactate, LPS and DAO in our study can be explained that even under similar challenges from IECs damage, patients with gut dysbiosis are more susceptible to compromised intestinal barrier integrity, bacterial translocation and sepsis, and subsequent worse outcome.

### The compromised intestinal barrier integrity in critically ill patients with pre-existing hyperglycemia

Previous studies have confirmed that gut dysbiosis is deeply associated with diabetes [27], and as the most specific symptom of diabetes, hyperglycemia has been proved to drive intestinal barrier dysfunction, impair intestinal barrier integrity, and cause bacterial translocation [12]. Nowadays, the cross-talks between diabetes, hyperglycemia, gut dysbiosis, intestinal barrier impairment, bacterial translocation, and systemic inflammatory response are gradually recognized [10, 11] and the compromised intestinal barrier integrity and increased bacterial translocation in patients with pre-existing hyperglycemia have been considered to cause worse prognosis in COVID-19 [14].

To further investigate the roles of compromised intestinal barrier integrity in critically ill patients with pre-existing hyperglycemia, we compared the intestinal barrier biomarkers and other clinical indicators between HbA1c < 6.5% Group and HbA1c ≥ 6.5% Group. The results showed that HbA1c ≥ 6.5% Group was lower in the NEU proportion, temperature, and lactate, indicating a milder severity of infection and a slighter disorder of circulatory system in this group. As is known to all, selection bias is almost inevitable in a retrospective observational study without a satisfactory sample size [28]. In the present study, selection bias resulted in a less severe infection and a milder circulatory dysfunction in HbA1c ≥ 6.5% Group. As mentioned above, the compromised intestinal barrier integrity did not accompanied with the damage of IECs. Compared with HbA1c < 6.5% Group, HbA1c ≥ 6.5% Group had similar levels of D-lactate and LPS but a lower level of DAO, which indicated that these patients suffered similar compromised intestinal barrier integrity even under a milder IECs damage. However, the indicators of disease severity and prognosis between these two groups displayed no statistical difference.

Taken together, Although the severities of infection, circulatory dysfunction, and IECs damage were milder, neither a slighter compromised intestinal barrier integrity nor a better outcome was achieved in HbA1c ≥ 6.5% Group. Since gut dysbiosis and compromised intestinal barrier integrity have been induced by hyperglycemia before the onset of critical illness [10, 12], for those patients with pre-existing hyperglycemia, the co-effect of hyperglycemia and critical illness will result in more severe compromised intestinal barrier integrity, bacterial translocation, and finally worse outcome [4, 5]. Therefore, we suggested that the compromised intestinal barrier integrity might be responsible for the poor prognosis in critically ill patients with pre-existing hyperglycemia.

### Perspective: the control of hyperglycemia in critical illness, to improve intestinal barrier integrity

Since the compromised intestinal barrier integrity plays an essential role in the development of critical illness, improving intestinal barrier integrity is considered to be a potential strategy in the treatment of critical illness [29]. As hyperglycemia can directly impair intestinal barrier and increase bacterial translocation [12], the control of hyperglycemia is thought to restore intestinal barrier integrity and inhibit bacterial translocation. Interestingly, some anti-diabetic agents with the capability of improving intestinal barrier integrity can bring advantages in the management of critical illness [30]. Given that hyperglycemia, irrespective of the diabetes status, is associated with poor prognosis in critically ill patients [4, 5], the role of compromised intestinal barrier integrity in critically ill patients with pre-existing hyperglycemia theoretically answers the question why it is important to control hyperglycemia.

This study had some limitations. First, although the compromised intestinal barrier integrity in patients with pre-existing hyperglycemia has been demonstrated in previous clinical studies [13, 31], the intestinal barrier function before the onset of critical illness were unavailable in our study. Second, HbA1c reflects the average blood glucose level in the past 2 to 3 months, the effect of blood glucose before 3 months is unknown. Besides, diabetic patients with well controlled hyperglycemia have not been discussed in the study. Third, as a retrospective observational study with small sample size, selection bias resulted in different severities of infection, circulatory dysfunction, and IECs damage between HbA1c < 6.5% Group and HbA1c ≥ 6.5% Group, and the conclusions of this study needs to be further confirmed in prospective studies.

## Conclusions

This was a retrospective observational study investigated the intestinal barrier biomarkers in critically ill patients. Results showed that D-lactate and LPS were associated with SOFA score and 90-day mortality, LPS was an independent risk factor of 90-day mortality, indicating that the intestinal barrier integrity was associated with the disease severity and prognosis in critical illness. In addition, DAO, NEU proportion, temperature, lactate were lower in HbA1c ≥ 6.5% Group while D-lactate, LPS, indicators of disease severity and prognosis showed no statistical difference between HbA1c < 6.5% Group and HbA1c ≥ 6.5% Group, indicating that although the severities of infection, circulatory dysfunction, and IECs damage were milder, neither a slighter compromised intestinal barrier integrity, nor a better outcome was achieved in patients with pre-existing hyperglycemia. Taken together, the compromised intestinal barrier integrity might be responsible for the poor prognosis in critically ill patients with pre-existing hyperglycemia.

## Data Availability

The raw data are available from the corresponding author on reasonable request.

## Abbreviations

AKI: Acute kidney injury
ALT: Alanine transaminase
APACHE II: Acute physiology and chronic health evaluation II
AST: Aspartate transaminase
CI: Confidence interval
CRP: C-reactive protein
DAO: Diamine oxidase
FBG: Fasting blood glucose
HbA1c: Hemoglobin A1c
HR: Hazard ratio
IECs: Intestinal epithelial cells
LOS: Length of stay
LPS: Lipopolysaccharide
MAP: Mean arterial pressure
NE: Norepinephrine
NEU: Neutrophil
RPM: Rate per minute
SOFA: Sequential organ failure assessment
WBC: White blood cell
